# Interest of the preventive and curative use of defibrotide on the occurrence and severity of sinusoidal obstruction syndrome after hematopoietic stem cell transplant in children

**DOI:** 10.1002/jha2.480

**Published:** 2022-05-31

**Authors:** Carl J. Rudebeck, Cécile Renard, Carine Halfon‐Domenech, Marie Ouachée‐Chardin, Michael Philippe, Frederic V. Valla, Yves Bertrand, Mathilde Penel‐Page

**Affiliations:** ^1^ Institut d'Hématologie et d'Oncologie Pédiatrique Hospices Civils de Lyon Lyon France; ^2^ University Claude Bernard ‐ Lyon I Lyon France; ^3^ Service de Réanimation et Soins Intensifs Pédiatriques ‐ Hôpital Femme Mère Enfant Hospices Civils de Lyon Lyon France

**Keywords:** children, defibrotide, grading classification, hematopoietic cell transplantation, preventive, veno‐occlusive disease/sinusoidal obstruction syndrome

## Abstract

Defibrotide (DF) is indicated for the treatment of severe sinusoidal obstruction syndrome (SOS) following hematopoietic stem cell transplantation (HSCT), but its prophylactic use against SOS is not recommended yet. This study describes the impact of the preventive and curative use of DF on reducing the incidence and severity of SOS in children. Patients aged 0–19 years, who received allogenic HSCT after myeloablative conditioning regimen with busulfan or total body irradiation in our comprehensive cancer center, between 2013 and 2017, were included. The Baltimore or modified Seattle criteria were used for SOS diagnosis. SOS was graded using the 2017 European Society for Blood and Marrow Transplantation classification defining severity criteria of SOS in children. SOS occurrence tended to decrease with prophylactic DF, but no significant difference was observed in terms of severity. When not treated with preventive DF, 50% (19/38) of the patients with SOS were graded severe to very severe, but only 37% (7/19) had organ dysfunction. Curative DF was administered at a median of 2 days post‐HSCT, for a median of 6.5 days. The absence of fatal SOS supports the use of early curative DF with acceptable toxicities and questions the optimal duration of DF treatment.

**Abbreviations**
BMT2: second bone marrow transplantation
CTCAE: Common Terminology Criteria for Adverse Events
DF: defibrotide
EBMT: European Society for Blood and Marrow Transplantation
GO: gemtuzumab‐ozogamicin
HSCT: hematopoietic stem cell transplantation
ICU: intensive care unit
IQR: interquartile range
MOF: multi‐organ failure
RT: refractory thrombocytopenia
S/VS: severe and very severe
SOS: sinusoidal obstruction syndrome
TBI: total body irradiation
VHR: very high‐risk
VS: very severe

## 1 INTRODUCTION

Sinusoidal obstruction syndrome (SOS) is a potentially life‐threatening complication following hematopoietic stem cell transplantation (HSCT) [1]. The mean incidence of SOS following HSCT is estimated around 15% (range 0%–62%) [2]. In the pediatric population, its incidence is higher, reaching approximately 20% [3]. The majority of SOS are mild or moderate, but 25% of cases are severe [4]. In most studies, severe SOS is defined as SOS with multiorgan failure (MOF). Before the use of defibrotide (DF), severe SOS was associated with a mortality rate exceeding 80% [2].

In SOS, sinusoidal endothelial damages associated with conditioning regimens lead to portal hypertension and hepatocyte dysfunction, eventually resulting in MOF [5]. The main clinical and biological signs include hepatomegaly, unexplained weight gain, ascites, rising bilirubin, and refractory thrombocytopenia (RT) [6]. Historically, the Baltimore and modified Seattle criteria were used to diagnose both adult and pediatric SOS, and the assessment of severity was performed using identical criteria regardless of the age of the patient. However, SOS in children presents with specific characteristics such as more frequent genetic predisposition, higher SOS incidence rate, higher late‐onset or anicteric SOS rate, and better results of DF treatment for SOS with MOF [6, 7]. To improve the pediatric management of SOS, the European Society for Blood and Marrow Transplantation (EBMT) recently proposed new guidelines defining specific diagnostic and severity criteria for SOS in children [6]. The SOS grading classification guides the initiation of therapeutic intervention according to 4 severity grades from mild to very severe (VS) [6].

Multiple well‐established risk factors for SOS have been identified, among which young age, inherited disorder such as osteopetrosis, pre‐existing liver disease, previous treatments with gemtuzumab‐ozogamicin (GO) or inotuzumab‐ozogamicin, conditioning regimen including busulfan or total body irradiation (TBI), and allogenic or repetitive HSCT [8, 9]. The recognition of these risk factors, the use of preventive treatments, and the early detection of SOS are key for an optimal management of the disease [6, 8, 9].

DF is the sodium salt of a single‐stranded polydeoxyribonucleotide derived from porcine tissue. It reduces procoagulant activity and increases fibrinolytic properties of stimulated endothelial cells [10]. DF is indicated in adult and children for the treatment of post‐HSCT severe SOS in Europe, and post‐HSCT SOS with pulmonary or renal dysfunction in the USA [11, 12]. Its use for SOS prophylaxis in clinical studies is promising in high risk patients but is not yet recommended [3].

This study reports the preventive and curative use of DF in a comprehensive cancer center between 2013 and 2017. The primary objective was to evaluate the impact of the preventive use of DF on the reduction of incidence and severity of SOS, by comparing very high‐risk (VHR) patients with prophylactic DF to a control group of VHR patients with no prophylactic DF. The secondary objectives were to describe the incidence and clinical course of SOS; report SOS severity using the 2017 EBMT grading classification; evaluate the contribution of the EBMT grading classification in routine practice; and appraise the clinical use and safety of DF.

## 2 METHODS

### 2.1 Patients and SOS prophylaxis

All patients aged from 0 to 19 years, who received allogenic HSCT after myeloablative conditioning with busulfan or TBI in our comprehensive cancer center (*Institut d'hématologie et d'oncologie pédiatrique*, IHOPe, Lyon, France) between 2013 and 2017, were included. Diseases leading to the transplantation were either malignant hematological diseases or nonmalignant hematological and immunological diseases. Data were obtained directly from a database (part of the European Bone Marrow Transplantation registry) including patients’ medical records. This study was conducted in agreement with local ethical requirements and declared to the French data protection agency (*Commission nationale de l'informatique et des libertés*, CNIL, reference: 2217016v0).

As per local guidelines, SOS prevention included ursodesoxycholic acid for all patients and DF when the risk of developing post‐HSCT SOS was very high. Patients were considered VHR when they received a second bone marrow transplantation (BMT2), were previously treated with GO or were transplanted for osteopetrosis [8, 9]. No patient received inotuzumab in this cohort. Patients receiving busulfan as part of their conditioning benefited from a very close pharmacological monitoring in order to maintain an adequate exposure (the area under the curve target was defined individually according to the underlying disease and the patient's risk factors) [13, 14]. Rigorous control of clinical, biological, and ultrasonographic features allowed prompt detection of SOS.

### 2.2 Criteria for the diagnosis and grading of SOS

The diagnosis of SOS was based on the Baltimore and modified Seattle criteria. SOS severity was graded using the 2017 EBMT classification (Table 1, ref. [6]). Grading criteria shared with other post‐HSCT complications were considered as related to SOS or not, according to the clinicians’ assessment.

**TABLE 1:** (Ref. (6)): European Society for Blood and Marrow Transplantation (EBMT) criteria for grading the severity of suspected hepatic SOS/VOD in children^a^

	Mild (grade 1)	Moderate (grade 2)	Severe (grade 3)	Very severe MOD/MOF (grade 4)
Liver function test^b^ (ALT, AST, GLDH)	≤2 × normal	>2 and ≤5 × normal	>5 x normal	
Persistent RT^b^	<3 days	3–7 days	>7 days	
Bilirubin (mg/dl)^b^, ^c^	< 2		≥2	
Bilirubin (μmol/L)	< 34		≥34	
Ascites^b^	Minimal	Moderate	Necessity for paracentesis (external drainage)	
Bilirubin kinetics				Doubling within 48 h
Coagulation	Normal	Normal	Impaired coagulation	Impaired coagulation with need for replacement of coagulation factors
Renal function GFR (ml/min)	89–60	59–30	29–15	<15 (renal failure)
Pulmonary function (oxygen requirement)	<2 L/min	>2 L/min	Invasive pulmonary ventilation (including continuous positive airway pressure)	
Central nervous system	Normal	Normal	Normal	New onset cognitive impairment

Abbreviations: ALT, alanine transaminase; AST, aspartate transaminase; CTCAE, common terminology criteria for adverse events; GFR, glomerular filtration rate; GLDH, glutamate dehydrogenase; MOD/ MOF, multi‐organ dysfunction/multi‐organ failure; RT, refractory thrombocytopenia; SOS/VOD, sinusoidal obstruction syndrome/veno‐occlusive disease.

^a^ If patient fulfills criteria in different categories, they must be classified in the most severe category. In addition, the kinetics of the evolution of cumulative symptoms within 48 h predicts severe disease.

^b^ Presence of ≥2 of these criteria qualifies for an upgrade to CTCAE level 4 (very severe SOS/VOD).

^c^ Excluding pre‐existent hyperbilirubinemia due to primary disease.

We considered impairment of coagulation as severe when antithrombin III level was <70% (as early reduction of antithrombin III may indicate the development of severe SOS [15] and a rate less than 70% leads to antithrombin III transfusion in our center), and as VS when prothrombin time was <50% or when the patient needed a replacement in coagulation factors [6].

### 2.3 DF: Administration, dose, duration, and toxicity

According to local guidelines, DF was started during conditioning when used preventively (median: 1 day before HSCT, interquartile range [IQR] [‐2–0.5]). It was then interrupted depending on clinical evolution. As a curative treatment, DF was started as soon as SOS worsened despite adequate symptomatic treatment with fluid and sodium restriction, or when SOS was associated with organ failure. It was maintained until SOS resolution, with no minimal duration of treatment. SOS resolution was defined as clinical and biological resolution of SOS criteria. In both its preventive and curative use, DF was given intravenously at 6.25 mg/kg every 6 h (25 mg/kg/day), in accordance with the recommendations [11].

Bleedings are common and feared side effects of DF. Hemorrhagic complications grade ≥2 according to the Common Terminology Criteria for Adverse Events (CTCAE), occurring under DF or before day 40 post‐HSCT for patients without DF treatment, were reported [16].

### 2.4 Statistical analyses

*p*‐values were obtained from a chi‐squared test or Fisher's exact test for categorical or dichotomous variables, and Mann–Whitney test for median values. A difference between two groups was considered significant when *p* was <0.05.

## 3 RESULTS

### 3.1 Patients

**FIGURE 1:**
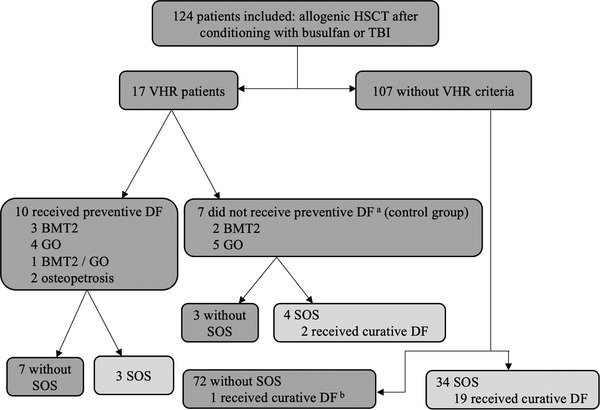
Patient flow chart. Abbreviations: BMT2, second bone marrow transplantation; DF, defibrotide; GO, gemtuzumab‐ozogamicin; HSCT, hematopoietic stem cell transplantation; SOS, sinusoidal obstruction syndrome; TBI, total body irradiation; VHR, very high‐risk. ^a^7 VHR patients did not receive prophylactic DF: two patients with BMT2 because of hemorrhagic complications; five patients previously treated with GO because of the modification of local guidelines in 2015. ^b^One patient received curative DF because of a suspected progressing SOS, but the treatment ended 2 days later when diagnosis was corrected as HHV6 reactivation

A total of 124 patients underwent myeloablative conditioning with busulfan or TBI between 2013 and 2017. Among them, 10 VHR patients received prophylactic DF: four for BMT2 including one recently treated with GO, four for recent treatment with GO, and two for osteopetrosis. Another seven VHR patients did not receive prophylactic DF. Two patients undergoing BMT2 and facing bleeding issues (after assessment of the benefit/risk balance by the referent physician), and five patients recently treated with GO, because of the modification of local guidelines. The 107 remaining patients were not considered VHR and did not receive preventive DF. The main characteristics of the patients from these three groups are described in Table 2. Overall, 32/124 (26%) patients received DF: 10 as a prophylactic treatment and 22 as a curative treatment of which one wrongly given (Figure 1).

**TABLE 2:** Patient characteristics

	No preventive DF (*n* = 114)		
	Non‐VHR patients (*n* = 107)	VHR patients (*n* = 7)	VHR patients with preventive DF (*n* = 10)
Age, years, median, (IQR)	7.3 (2.8–13.5)	12.1 (7.8–16.2)	13.5 (2–15.2)
Gender			
Male	65 (61%)	6 (86%)	8 (80%)
Primary disease			
Malignant	68 (64%)	6 (86%)	7 (70%)
Leukemia^a^	60	6	7
Other^b^	8	0	0
Non‐malignant	39 (36%)	1 (14%)	3 (30%)
Immune deficiency	19	1	0
RBC diseases^c^	16	0	0
Other^d^	4	0	3
Type of donor			
Matched‐related donor	36 (34%)	1 (14%)	3 (30%)
Matched unrelated donor	27 (25%)	5 (72%)	2 (20%)
Haplo‐identical family donor	2 (2%)	0	0
Mismatched unrelated donor	42 (39%)	1 (14%)	5 (50%)
Origin of graft			
Bone marrow	82 (77%)	6 (86%)	7 (70%)
Peripheral blood stem cells	4 (4%)	1 (14%)	1 (10%)
Umbilical cord blood	21 (19%)	0	2 (20%)
Conditioning drugs			
Myeloablative drugs			
Busulfan	81 (76%)	5 (71%)	9 (90%)
TBI	26 (24%)	2 (29%)	1 (10%)
Immunosuppression			
Ciclosporin	107 (100%)	7 (100%)	10 (100%)
Antithymocyte globulin	81 (76%)	6 (86%)	5 (50%)
SOS risk factors (unrelated to the characteristics above)			
Previous stem cell transplantation	–	2 (29%)	4 (40%)
Previous treatment with GO	–	5 (71%)	5 (50%)
Osteopetrosis	–	0	2 (20%)
Abnormal hepatic workup	27 (25%)	3 (43%)	5 (50%)

Results are presented as n (%) or median (IQR) when relevant.

Abbreviations: DF, defibrotide; GO, gemtuzumab‐ozogamicin; IQR, interquartile range; RBCs, red blood cells; SOS, sinusoidal obstruction syndrome; TBI, total body irradiation; VHR, very high‐risk.

^a^ Leukemia (acute lymphoblastic and myelogenous leukemia, biclonal leukemia, juvenile myelo‐monocytic leukemia, chronic lymphocytic leukemia).

^b^ Other (lymphoma, myelodysplasic syndrome, combined myelodysplasic syndrome/severe aplasic anemia).

^c^ RBC diseases (sickle cell disease, thalassemia, Blackfan diamond disease).

^d^ Other (familial haemophagocytic lymphohistiocytosis, osteopetrosis, adrenoleukodystrophy).

### 3.2 Preventive use of DF and VHR patients

Among the VHR patients treated with preventive DF, 30% developed SOS (3/10) compared to 57% in the control group (4/7, *p* = 0.349).

Concerning the three patients who developed SOS after prophylactic DF, one was undergoing BMT2, and two were previously treated with GO. One of them received DF until day 20 post‐HSCT. On day 21, bilirubin raised from 31 to 95 μmol/L with significant weight gain: DF was reintroduced for 10 more days leading to the immediate resolution of symptoms. All VHR patients diagnosed with SOS and treated with prophylactic DF were graded severe (3/3). In the control group, the four SOS were graded mild to severe (BMT2: mild SOS, *n* = 1; previous GO: mild SOS, *n* = 1; moderate SOS, *n* = 1; severe SOS, *n* = 1). The two patients graded moderate or severe required curative DF, and treatment was initiated 1 and 2 days after SOS diagnosis, respectively. None of the VHR patients with SOS had organ dysfunction, were transferred to intensive care unit (ICU), or died because of SOS (Table 3).

**TABLE 3:** Incidence, severity, and clinical course of SOS

	All patients; *n* = 124	VHR patients without preventive DF; *n* = 7	VHR patients with preventive DF; *n* = 10
SOS incidence, *n* (%)	*n* = 41 (33%)	*n* = 4 (57%)	*n *= 3 (30%)
Median time between HSCT and SOS diagnosis, days (IQR)	10 (7–13)	11.5 (9.75–13)	7 (7–14.5)
ICU transfer (≤ +100 days post‐HSCT)			
All	8 (20%)	0	1 (33%)
Because of SOS	4 (10%)	0	0
Because of DF‐related adverse events	0	0	0
Death (≤ +1‐year post‐HSCT)			
All	8 (20%)	0	2 (66%)
Because of SOS	0	0	0
Because of DF‐related adverse events	0	0	0

Abbreviations: DF, defibrotide; EBMT, European society for blood and marrow transplantation; HSCT, hematopoietic stem cell transplantation; ICU, intensive care unit; IQR, interquartile range; SOS, sinusoidal obstruction syndrome; VHR, very high‐risk.

### 3.3 SOS incidence, severity grading, and clinical course

Overall, the incidence of SOS in this study was 33% (41/124), with a median diagnosis at 10 days post‐HSCT (IQR 7–13). Eight patients with SOS (20%) were transferred to ICU before day +100 (four due to SOS, four due to other complications). One‐year overall survival among patients with SOS was 80% (33/41). None of them died because of SOS (Table 3).

Excluding the patients treated with preventive DF, SOS severity according to the EBMT classification, was mild in 29% of patients (11/38), moderate in 21% (8/38), severe in 21% (8/38), and VS in 29% (11/38). The grading of the patients as severe SOS (8/8) was based on the presence of biological factors likely to be associated with MOF, but these patients did not present with organ dysfunction (Table 4A). Among VS SOS patients, 64% (7/11) had at least one organ dysfunction, but 36% (4/11) had isolated biological factors likely to be associated with MOF (Table 4B). The incidence of organ dysfunction and/or failure was 18% (7/38) in patients with SOS who were not treated with preventive DF. This incidence reached 37% (7/19) when focusing on severe and VS (S/VS) SOS. Among these patients, 55% received curative DF (21/38). Two patients with severe (*n* = 1) or VS SOS (n = 1) did not receive curative DF because of the favorable evolution of the disease following **fluid** restriction and diuretics.

**TABLE 4A:** Characteristics of severe patients

	Severe SOS (*n* = 8)
Biological factors likely to be associated with MOF or death	
Persistent RT > 7 days	2 (25%)
Bilirubin (μmol/L) ≥34	3 (38%)
Impaired coagulation (antithrombin III < 70%)	7 (88%)
Organ dysfunction or failure	
AST, ALT > 5 x normal	0
Ascites requiring paracentesis (external drainage)	0
Renal function GFR: 29–15 ml/min	0
Noninvasive or invasive pulmonary ventilation	0
Patients graded severe because of biological factors without organ dysfunction	8 (100%)
Patients graded severe with organ dysfunction or failure	0

Abbreviations: ALT, alanine transaminase; AST, aspartate transaminase; DF, defibrotide; GFR, glomerular filtration rate; MOF, multi‐organ failure; PT, prothrombin time; RT, refractory thrombocytopenia; SOS, sinusoidal obstruction syndrome; VS, very severe.

**TABLE 4B:** Characteristics of VS patients

	VS SOS (*n* = 11)
Biological factors likely to be associated with MOF or death	
Persistent RT > 7 days	9 (82%)
Bilirubin (μmol/L) ≥34	11 (100%)
Doubling bilirubin within 48h	5 (45%)
Organ dysfunction or failure	
AST, ALT > 5 x normal	4 (36%)
Ascites requiring paracentesis (external drainage)	1 (9%)
Impaired coagulation with need for replacement of coagulation factors or PT < 50%	5 (45%)
Renal function GFR: < 15 ml/min; dialysis dependence	2 (18%)
Noninvasive or invasive pulmonary ventilation	1 (9%)
New onset of cognitive impairment	0
Patients graded VS because of biological factors without organ dysfunction	4 (36%)
Patients graded VS with organ dysfunction or failure	7 (64%)

Abbreviations: ALT, alanine transaminase; AST, aspartate transaminase; DF, defibrotide; GFR, glomerular filtration rate; MOF, multi‐organ failure; PT, prothrombin time; RT, refractory thrombocytopenia; SOS, sinusoidal obstruction syndrome; VS, very severe.

### 3.4 DF: Administration, dose, duration, and toxicity

For patients without DF prophylaxis, the median time until curative DF after SOS diagnosis was 2 days (IQR 1–4). The median duration of full dose DF treatment was 6.5 days (IQR 5–13) with a median of 4 extra days (IQR 2–4) before DF was fully stopped (progressive dose reduction). The full dose of curative DF required was significantly longer for patients with VS SOS (median duration of 14 days [IQR 8–14]) than for those with moderate and severe SOS (5 days [IQR 3.75–6]; *p* = 0.002). For patients receiving preventive DF and not developing SOS, the median duration of full dose DF post‐HSCT was 19.5 days (IQR 18.25–20.5) with a median of 4 extra days (IQR 3.75–4) before DF was fully stopped.

**FIGURE 2:**
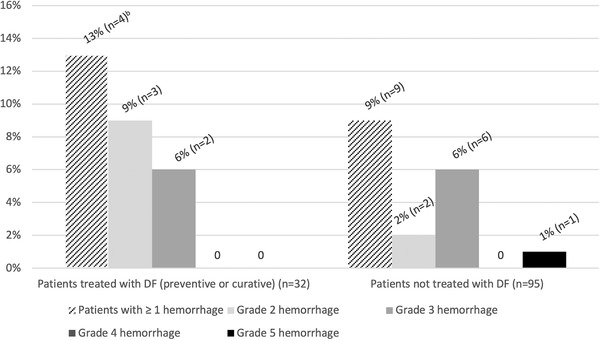
Hemorrhagic adverse events. DF, defibrotide. ^a^Hemorrhagic complications were graded according to the Common Terminology Criteria for Adverse Events (CTCAE). ^b^One patient had two distinct hemorrhages of grade 3 and 4, thus for the four patients in the group treated with DF, a total of five significant hemorrhages were considered

Significant bleeding was found in 13% (4/32) of patients treated with DF and in 9% (9/95) of patients without DF (*p* = 0.736; Figure 2). Two patients had to stop DF because of bleeding. In one case, it concerned a patient with curative DF who had uncontrolled gynecologic hemorrhages (grade 3). The other case concerned a patient with prophylactic DF who presented with intraparenchymal and intraventricular cerebral hemorrhage (grade 2), after SOS was diagnosed. No fatal bleeding occurred in patients treated with DF.

## 4 DISCUSSION

### 4.1 Preventive use of DF and VHR patients

The incidence of SOS tended to be lower in VHR patients who received preventive DF compared to VHR patients without preventive DF. The use of preventive DF for reducing post‐HSCT SOS incidence has already been suggested in several studies, among which a phase 3 randomized controlled trial [17, 18]. Although this result did not reach significance in the present study, likely due to insufficient statistical power, other observations support this trend. First, for one patient the appearance and resolution of SOS was related to the discontinuation and reintroduction of DF treatment. Second, the two patients grafted for osteopetrosis who received prophylactic DF did not declare SOS despite a reported SOS incidence of 60% in this specific population [19]. No clear reduction in SOS severity was observed in patients receiving preventive DF. Although more severe SOS were diagnosed in the prophylactic DF group compared to the control group (3/3 vs. 1/4), none had organ dysfunction, were transferred to ICU, or died because of SOS. No life‐threatening or fatal SOS was observed in the control group either, possibly due to the early initiation of curative DF [20]. Larger prospective studies are needed to better define the impact of prophylactic DF on SOS and further characterize the population, which could be eligible for DF prophylaxis, by paying particular attention to the benefit of DF prophylaxis compared to early curative DF.

### 4.2 SOS incidence, severity grading, and clinical course

No late‐onset SOS was diagnosed herein, although some studies in children have described that 15%–20% of SOS cases appear beyond day +30 post‐HSCT [6, 21]. The retrospective collection of data may have impaired its detection, particularly since the Baltimore and modified Seattle criteria were used, and SOS diagnosis is allowed only before day 21 post‐HSCT in these former classifications [22, 23]. However, no SOS was severe enough after day +30 to be mentioned in any medical report.

Herein, SOS grading in patients who were not treated with preventive DF showed S/VS SOS in half of the patients compared to approximately 25% of severe SOS described in the literature before the 2017 EBMT grading classification [4]. This can be explained by the fact that in the new classification, SOS severity includes early predictive signs of poor prognosis, whereas severe SOS used to be linked to the presence of MOF [2, 6].

Given the high number of patients graded S/VS, we decided to split the new severity criteria into two parts: one gathering the early biological signs likely to be associated with MOF or death due to SOS [6, 8, 15, 24], and a second including significant signs of recognized organ dysfunction. By dividing EBMT grading criteria, only about a third of patients with S/VS SOS were found to have recognized organ dysfunction. The redefinition by EBMT of the severity criteria aimed at improving the management of patients with SOS, offering the potential for an early therapeutic intervention to prevent the evolution of SOS toward organ dysfunction [6]. Indeed, early therapeutic intervention with DF is predictive of SOS resolution [20]. Our comprehensive cancer center has taken this into account, as demonstrated by the short delay between SOS diagnosis and the start of curative DF. This could explain the low rate of organ dysfunction described above, together with the low rate of ICU transfer and the absence of death related to SOS. Thus, the use of the EBMT grading classification could have a strong impact on SOS evolution by homogenizing the prompt initiation of DF. In accordance with the literature, this observation supports the superiority of early DF initiation to prevent SOS evolution toward death [20, 25].

However, among patients with S/VS SOS, two had a favorable evolution without curative DF. One was graded S/VS due to hyperbilirubinemia, and the second due to the association of hyperbilirubinemia, RT, and hepatic dysfunction. Using the EBMT grading classification, these patients would have been treated with DF, whereas disease evolution was favorable with fluid restriction and diuretics. Although no other cause than SOS was found to explain these biological disorders, it remains uncertain whether or not they were related to SOS (especially hepatic dysfunction) considering the quick resolution of the disease. The biological factors related to SOS (especially hyperbilirubinemia and RT) are easily found in common complications post‐HSCT such as infections, graft‐versus‐host disease, drug toxicity, or vascular endothelial syndromes. The combination of SOS with these complications could mislead its grading and lead to DF implementation based on biological signs, which are not specific. Combining clinical evaluation with biological signs and ultrasonography remains fundamental to start DF treatment.

### 4.3 DF: Administration, dose, duration, and toxicity

According to recommendations, curative DF should be administered for a minimum of 21 days [11]. A recent paper highlighted the lack of compelling reason to continue DF for a predetermined length of time in patients achieving early resolution of SOS symptoms [26]. The present study supports this consideration as the median time with a full dose of curative DF was a little less than a week. Only two patients of 21, both graded VS, received DF for more than 21 days. The other 19 discontinued DF earlier when SOS symptoms resolved, without SOS relapse. These data challenge the minimal duration of curative DF, which may be adapted to the evolution of SOS. Herein, the median time with a full dose of preventive DF in patients not developing SOS was just under 3 weeks compared to the regular 30 days post‐HSCT [17]. One patient, previously treated with GO, developed SOS 1 day after the discontinuation of prophylactic DF. However, prompt reintroduction of DF led to SOS resolution with no serious consequences. Larger prospective studies are needed to define the optimal duration of preventive DF for each situation, but here again, early use of DF seemed to prevent the poor evolution of SOS.

The main complication of DF is hemorrhage. No significant difference was observed for hemorrhagic events between patients treated with DF and those not treated. No toxic death due to DF was reported here. The present data show that the safety profile in terms of bleeding is similar to that reported in previous studies [17, 27]. However, two patients had to stop DF due to significant bleeding (grade 2 cerebral bleeding and grade 3 genital bleeding).

### 4.4 Strengths and limitations

Among the key strengths of this study are the homogeneity in terms of SOS management, the real‐life testing of the 2017 EBMT grading classification for SOS, and the consistency of the results with larger studies for an impact of preventive DF in a selected pediatric population and, more so, for the impact of early DF administration on SOS outcomes [17, 20, 25]. Moreover, the short DF treatment duration associated with the absence of death due to SOS is an interesting observation that could be explored in larger studies.

Acknowledged limitations include the small sample size of patients receiving preventive DF, the use of antithrombin III level as a severity criterion of SOS, which could have overestimated the incidence of S/VS SOS, and the use of the Baltimore and modified Seattle criteria to diagnose SOS, which are no longer recommended and could bias SOS incidence and the derived results (S/VS SOS proportion, DF treatment duration).

## 5 CONCLUSION

The present study shows that despite a tendency toward a decreased incidence of SOS after prophylactic DF, no difference is observed in terms of severity. The retrospective grading of the disease in patients not treated with preventive DF found a high proportion of S/VS SOS according to the 2017 EBMT grading classification. Most of these patients had isolated biological criteria predicting a potentially poor evolution of the SOS but had no organ dysfunction. These criteria are key for the early initiation of curative DF but are shared with multiple post‐HSCT complications. Even if an overall evaluation of the patient remains fundamental before deciding to start DF, EBMT grading classification might facilitate its early implementation. The low rate of organ dysfunction and the absence of fatal SOS evolution in this study support the superiority of the early initiation of curative DF with acceptable toxicities but challenge its minimal duration. Prospective studies are needed to confirm these results and better define which patients would benefit from prophylactic DF rather than the early use of curative DF.

## CONFLICT OF INTEREST

The authors have no competing financial interest in relation to the work described.

## AUTHOR CONTRIBUTIONS

C. J. Rudebeck, M. Penel‐Page, and Y. Bertrand designed the study. C. J. Rudebeck and M. Penel‐Page collected the data. C. J. Rudebeck, M. Penel‐Page, and Y. Bertrand analyzed the data. C. J. Rudebeck, M. Penel‐Page, and Y. Bertrand analyzed and interpreted the results. C. J. Rudebeck and M. Penel‐Page wrote the manuscript. F. V. Valla, C. Renard, C. Halfon‐Domenech, M. Philippe, and M. Ouachée‐Chardin revised the manuscript. All authors significantly contributed to the study and reviewed the final version of the manuscript.

## Data Availability

Data were obtained directly from a database (part of the European Bone Marrow Transplantation registry) including patients’ medical records. This study was conducted in agreement with local ethical requirements and declared to the French data protection agency (Commission nationale de l'informatique et des libertés, CNIL, reference: 2217016v0).
